# Banded Versus Non-banded Sleeve Gastrectomy: A Systematic Review and Meta-Analysis

**DOI:** 10.7759/cureus.52799

**Published:** 2024-01-23

**Authors:** Abdulkreem Al-Juhani, Galal F Sharaf, Eman M Alyaseen, Abdullah Alkurdi, Ammar S Azhari, Saleh Hussain Alshaiban, Abdulelah A Otaif, Abdullah W abumadian, Alaa J Alshawi, Yara A Aldarami

**Affiliations:** 1 Surgeon, King Abdulaziz University Faculty of Medicine, Jeddah, SAU; 2 General Surgery, University of Queensland, Cairo, EGY; 3 Medicine and Medical Science, Arabian Gulf University, Manama, BHR; 4 Medicine, AL-Rayan Colleges, Madina, SAU; 5 Medicine, King Abdulaziz University, Jeddah, SAU; 6 Medicine, Najran University, Najran, SAU; 7 Medicine, Jazan University, Jazan, SAU; 8 Medicine, King Abdulaziz University Hospital, Jeddah, SAU; 9 Medicine, Ibn Sina National College For Medical Studies, Jeddah, SAU; 10 Medicine, King Khalid University, Abha, SAU

**Keywords:** lbsg, systematic review and meta analysis, bariatric surgery, laparoscopic sleeve gastrectomy, laparoscopic band sleeve gastrectomy

## Abstract

Standard bariatric surgeries include biliopancreatic diversion (BPD), sleeve gastrectomy (SG), Roux-en-Y gastric bypass (RYGB), and adjustable gastric banding (AGB). Laparoscopic sleeve gastrectomy (LSG) is currently favored due to safety, efficacy, and shorter operation time. However, previous literature shows 75.6% weight regain post LSG. Introducing Laparoscopic band sleeve gastrectomy (LBSG) to maintain pouch size is proposed to improve outcomes and reduce weight regain. This study aims to compare the safety and efficacy of LSG vs. LBSG in obese patients. A comprehensive search strategy was executed to identify pertinent literature comparing LBSG and LSG in obese patients. Eligible studies underwent independent screening, and pertinent data were systematically extracted. The analysis employed pooled risk ratios (RR) for dichotomous outcomes and mean differences (MD) for continuous variables, each accompanied by their respective 95% confidence intervals (CI). Our systematic review and meta-analysis included 15 studies encompassing 3929 patients. Regarding body mass index (BMI), at six, 12, and 24 months, no substantial differences were found between LBSG and LSG groups (p < 0.05). Still, at 36 months, LBSG exhibited significantly lower BMI than LSG (MD = -2.07 [-3.84, -0.29], p = 0.02). Excess Weight Loss (EWL) favored LBSG at 12, 24, and 36 months with MD of 3.30 [0.42, 6.18], 4.13 [1.44, 6.81], and 18.43 [9.44, 27.42], p = 0.02, 0.003, < 0.00001, respectively). Operative time did not significantly differ between the procedures (MD = 2.95, 95%CI [-0.06, 5.95], p = 0.05). Resolution of comorbidities, overall complications, post-operative bleeding, reflux, and early complications did not significantly differ between LBSG and LSG. However, LBSG showed higher post-operative regurgitation than LSG (RR = 2.38, 95%CI [1.25, 4.54], p = 0.008). LBSG showed a substantial decrease in BMI at three-year follow-up and higher EWL at one, two, and three years. However, LBSG procedures exhibited a higher incidence of post-operative regurgitation symptoms than LSG. No substantial differences were noted in BMI at six, 12, or 24 months, EWL at six months, operative time, bleeding, reflux, or overall complications.

## Introduction and background

Obesity is a multifactorial disease that is linked to several comorbidities, mainly cardiovascular diseases and type 2 diabetes, and is associated with increased mortality rates [1]. The obesity rate has dramatically increased in both sexes, across all ages, with the highest rate in the elderly and females [1]. The exact mechanism of obesity remains controversial but is believed to be related to a complex interaction between factors such as regulation of energy balance, appetite, and physical activity besides hereditary and environmental factors [2]. Adopting lifestyle changes continues to serve as the fundamental approach to addressing obesity, with consideration given to body mass index (BMI) thresholds. When BMI reaches or exceeds 30, pharmaceutical interventions can assist in achieving weight loss. However, individuals with a BMI surpassing 40 or those with a BMI exceeding 35, coupled with concurrent health conditions, are advised to consider bariatric surgery as the recommended course of action [3,4].

Standard bariatric surgeries are Biliopancreatic diversion (BPD), sleeve gastrectomy (SG), Roux-en-Y gastric bypass (RYGB), and adjustable gastric banding (AGB) [5]. While the optimal procedure remains a subject of debate, the Laparoscopic sleeve gastrectomy (LSG) is currently the prevailing choice in practice [6]. This preference stems from its established safety profile, efficacy, and notably shorter duration in the operating room [6].

Extended follow-up studies reveal a significant incidence of long-term weight regain, affecting approximately 75.6% of patients six years post LSG. This aspect notably constitutes a primary drawback of this procedure. Consequently, introducing a laparoscopic band sleeve gastrectomy (LBSG) to maintain the gastric pouch's size has been hypothesized as a means to enhance surgical outcomes and mitigate the post-operative weight regain, akin to the principles observed in banded RYGB [7,8].

Multiple clinical trials showed that the banded procedure has several benefits, including reduced food intake, delayed gastric emptying, altered hormonal levels, and esophageal peristalsis. Besides weight loss, less weight regain, reduction of reflux symptoms, and resolution of comorbidities [9,10]. The superiority between banded and non-banded procedures remains a key inquiry in this field. Although a previous meta-analysis sought to address this query, its findings were constrained by several limitations, including limited incorporated randomized controlled trials (RCTs), small sample sizes, and insufficient follow-up durations. Thus, further investigations were warranted to provide more comprehensive insights into this matter [11]. Our study aims to comprehensively compare the safety and efficacy of LSG vs. LBSG in obese patients.

## Review

Methods

This meta-analysis followed the preferred reporting item for systematic reviews and meta-analysis (PRISMA) guidelines and implemented the guidelines of the Cochrane Handbook for Systematic Reviews of Interventions [12,13].

Literature Search

We extensively searched various electronic databases, including Cochrane Library, PubMed, Scopus, Web of S.

cience, and EMBASE. Two reviewers independently searched Each database using the following research key terms: (Band OR Banded OR “Non-Banded” OR Banding OR “Silastic Band”) AND (Gastrectom* OR “Stomach stapling” OR “Gastric sleeve surgery”) AND (Bariatric OR Metabolic OR Obes*). This extensive search was conducted from inception till January 2024. Additionally, reference lists of eligible articles and previous meta-analyses were examined manually to identify relevant citations.

Eligibility Criteria

Two reviewers evaluated the retrieved studies independently and meticulously examined their eligibility according to our predetermined criteria: 1) We included studies on obese adults (>18 years old) with BMI > 35 kg/m^2^). 2) Studies comparing LBSG vs. LSG. 3) Studies assessed any of the following outcomes for the mentioned interventions: BMI, EWL, operative time, reflux, regurgitation, or complications.

Numerous studies were excluded from analysis due to our exclusion criteria: 1) non-comparative studies, 2) studies not published in English, 3) editorial letters, abstracts, or comments only, and 4) incorporation of unpublished data.

Our primary outcome was to compare the improvement of anthropometric parameters after laparoscopic banded versus non-banded sleeve gastrectomy (BMI and percentage of Excess weight loss (EWL) after six, 12, 24, and 36 months of follow-up). The secondary outcomes were operative time, post-operative complications, bleeding, reflux, regurgitation, and resolution of comorbidities.

Data Gathering

data were extracted in offline data extraction sheets. Extracted data including various aspects such as the study ID, publication year, study location, study arms, sample size, gender distribution, participant ages, study inclusion criteria, conclusions, primary and secondary outcomes (anthropometric parameters, operative time, post-operative complications, bleeding, reflux, and regurgitation). Data in formats (such as median or range) were translated to mean ±SD using Cochrane Handbook Standard Deviation guidelines [10].

Risk of Bias Assessment

The quality assessment of the included trials was performed using the Cochrane Risk of Bias assessment tool 1 (ROB1) (designed for interventional studies) [14]. It includes the following Items: random sequence generation, allocation concealment, blinding of investigators and participants, attrition bias, selective reporting, and other biases. Each domain was meticulously assessed to evaluate potential biases within the included studies comprehensively. The quality assessment of our included cohort studies was judged by the National Institutes of Health (NIH) tool [15]. Also, we used MINORS Criteria for the assessment of non-randomized clinical trials, which encompass the following seven domains: study aim, consecutive patient inclusion, prospective data collection, an appropriate endpoint to aim, evaluation of endpoint, follow-up period and percentage of loss of follow up [16].

Data Synthesis

We utilized different statistical methods based on outcome types: mean differences (MD) with standard deviations were pooled for continuous outcomes, while risk ratios (RR) with 95% confidence intervals were employed using the Mantel-Haenszel method for dichotomous outcomes. A fixed-effect model was initially used for homogeneous studies, but a random-effects model was applied for heterogeneous ones. Statistical heterogeneity was evaluated through I2 and Chi2 tests, with a p-value < 0.10 indicating heterogeneity and I2 ≥ 50% suggesting high heterogeneity. We used Review Manager (RevMan) version 5.4 to conduct all the analyses.

Results

Literature Search Results

**Figure 1:**
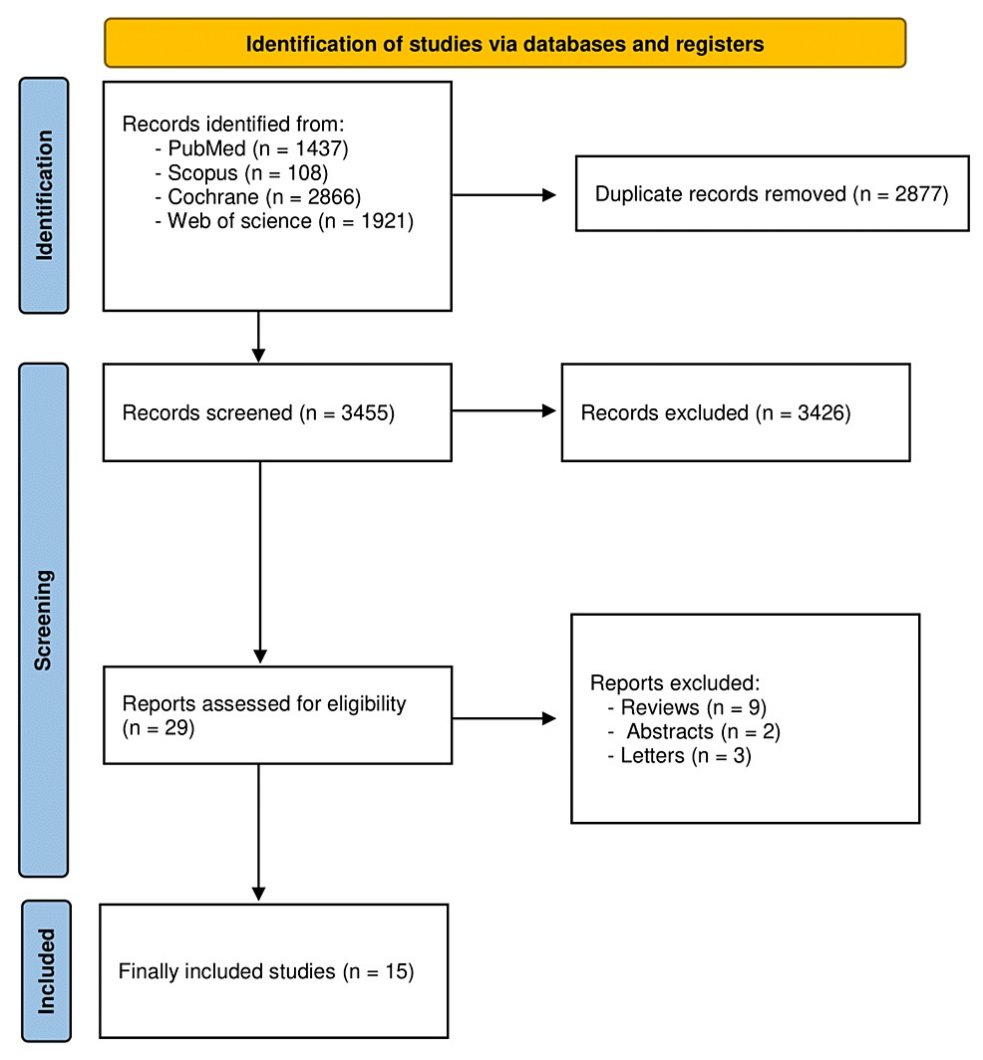
PRISMA flow chart

Following an initial search across our five databases, 6,332 papers met our inclusion criteria; after 2,877 duplicate papers were removed, we had a total of 3,984 articles available for the title and abstract screening. We applied our inclusion and exclusion criteria, resulting in 29 articles available for full-text examination. Finally, we had 15 studies [10,17-30] included in this systematic review meta-analysis. The PRISMA diagram depicting this selection process is shown in Figure 1.

Characteristics of the Included Studies

We have fifteen included studies encompassing a total of 3929 patients. Regarding geographical distribution, seven were conducted in Egypt, four in Germany, two in Italy, and the remaining in India and Belgium. In our study, 3,122 patients were subjected to LSG procedure while only 807 LBSG, estimated BMI ranged from 45 to 53 kg/m^2^, with the majority of participants were hypertensive and diabetic. Patients’ characteristics and a summary of the included studies are provided in Table 1.

**Table 1:** Summary and baseline characteristics of the included studies Abbreviations: LSG= Laparoscopic Sleeve Gastrectomy; LBSG= Laparoscopic Band Sleeve Gastrectomy; RCT= Randomized clinical trial; BMI= Body Mass Index; DM= Diabetes mellitus; HTN= Hypertension; OSAS=obstructive sleep apnea syndrome; EWL= Excess weight loss

S. No	Study	Study arms, n(%)	Site	Study design	Age, (mean±SD)y	Male, n(%)	Follow-up duration (Years)	BMI(kg/m2), (mean±SD)	Comorbidities, n(%)	Inclusion criteria	Primary endpoints	Conclusion
1	Afifi et al. 2022 [19]	LBSG, 20(50)	Egypt	Retrospective cohort study	35.9 ± 7.2	10(50)	Up to Three	45.2 ± 4.25	1. DM, 8(40) 2. HTN, 6(30) 3. HTN and DM, 0	1. Patients undergoing either BSG or NBSG 2. From January 2018 to December 2020 3. All patients above 18 years old 4. Patients were followed at 3, 6, 12, 24, and 36 months at clinics	1. BMI 2. Resolution of Comorbidities	"This study suggests that BSG is more effective than NBSG in reducing BMI at 36 months. Future large-sample randomized trials are needed to compare the two surgeries regarding long-term postoperative complications and the need for a secondary operation. The authors recommend testing the application of the band around the upper third of the remaining stomach in reducing the postoperative reflux after banded sleeve gastrectomy."
		LSG, 20(50)			37.2 ± 8.7	10(50)		44.7 ± 4.49	1. DM, 6(30) 2. HTN, 2(10) 3. HTN and DM, 6(30)			
2	Albalkiny et al. 2022 [18]	LBSG, 49(49.49)	Egypt	Prospective Cohort Study	42.5 ± 9.8	22(45)	Up to Three	41 ± 4.5	1. DM, 23(47) 2. HTN, 30(61) 3. Apnea, 20(40) 4. Dyslipidemia, 32(65) 5. GERD, 21(42.8)	1. Patients who underwent LSG 2. Between January 2015 and January 2018 3. All patients above 18 years old 4. With a BMI more than 40 or 35 with at least one comorbidity 5. Had given written informed consent	1. EWL (%) 2. Weight Regain	"LBSG using PTFE is superior to LSG in promoting and maintaining short-term and mid-term weight loss without adding extra burden in terms of postoperative complications."
		LSG, 50(50.51)			43 ± 1	20(40)		41 ± 4.6	1. DM, 20(40) 2. HTN, 33(66) 3. Apnea, 18(36) 4. Dyslipidemia, 35(70) 5. GERD, 23(46)			
3	Fink et al. 2023 [17]	LBSG, 47(50)	Germany	Prospective Cohort Study	43.3 ± 9.196	12(25.5)	Up to Five	51 ± 6.64	1. DM, 11(23.4) 2. HTN, 26(53.3) 3. GERD, 7(14.9) 4. Hiatal hernia, 9(19.1) 5. Esophagitis, 11(23.4)	1. Age between 18 and 65 years 2. History of conservative weight-loss treatment failure 3. With a BMI more than 40 or 35 with at least one comorbidity	1. EWL (%) 2. Post-operative complications	"Five-year weight loss after BSG was 9% EWL and 4.2% TWL higher compared to SG. The main added morbidity following BSG was postprandial regurgitation."
		LSG, 47(50)			40.9 ± 9.03	16(34)		50.7 ± 6.47	1. DM, 6(12.8) 2. HTN, 20(42.6) 3. GERD, 7(14.9) 4. Hiatal hernia, 6(12.8) 5. Esophagitis, 6(12.8)			
4	Fouly et al. 2022 [21]	LBSG, 50(50)	Egypt	Non-RCT	36.72 ± 8.45	24(48)	Up to Five	45.14 ± 5.04	1. DM, 25(50) 2. HTN, 7(14) 3. HTN and DM, 0(0)	1. Age >18 and <65y 2. History of conservative weight-loss treatment failure 3. With a BMI more than 40 or 35 with at least one comorbidity	Changes in BMI over five follow-up	"BLSG may have favourable outcomes than NLSG, and this effect persisted without weight regain."
		LSG, 50(50)			36.96 ± 9.43	17(34)		44.42 ± 4.87	1. DM, 15(30) 2. HTN, 2(4) 3. HTN and DM, 13(26)			
5	Hany et al. 2022 [22]	LBSG, 132(9.48)	Egypt	Retrospective cohort study	33.83 ± 9.74	28(21.21)	Up to Four	47.5 ± 7.12	1. HTN, 40(30.3) 2. DM, 24(18.2) 3. OSA, 19(14.4) 4. Dyslipidemia, 40(30.3) 5. Osteoarthritis, 34(25.8) 6. CD, 8(6.1) 7. Psych. disorders, 16(12.1) 8. Vascular diseases, 20(15.2)	1. Adult patients who underwent laparoscopic banded or non-banded SG 2. Between January 2016 and December 2017 3. With a BMI more than 40 or 35 with at least one comorbidity 4. Had given written informed consent	1. Post-operative morbidity 2. Post-operative endoscopic findings 3. Post-operative laboratory parameters	"Although the percentage of weight loss achieved in the BSG group was low in the first year postoperatively, the mid-term (sustained) weight loss associated with BSG was superior to that associated with non-banded SG. BSG is a safe Procedure with no significant mid-term band-related morbidity; its impact on the resolution of comorbidities is equivalent and perhaps superior to SG."
		LSG, 1260(90.52)			34.67 ± 10.39	321(25.476)		47.43 ± 6.976	1. HTN, 381(30.2) 2. DM, 218(17.3) 3. OSA, 160(12.7) 4. Dyslipidemia, 379(30.1) 5. Osteoarthritis, 322(25.6) 6. CD, 80(6.3) 8(6.1) 7. Psych. disorders, 162(12.9) 8. Vascular diseases, 191(15.2)			
6	Hany et al. 2022 (2) [30]	LBSG, 132(9.35)	Egypt	Retrospective cohort study	34.38 ± 9.71	28(21.2)	Up to Four	47.69 ± 7.13	1. HTN, 387(30.3) 2. DM, 221(17.3) 3. SA, 161(12.6) 4. Dyslipidemia, 386(30.2) 5. OA, 328(25.6) 6. CD, 81(6.3) 7. Psych. disorders, 162(12.7) 8. Vascular diseases, 194(15.2)	1. All consecutive patients who had primary LSG or BSG 2. Between January 2016 and December 2017 3. Completed a follow-up period of 4 years 4. With a BMI more than 40 or 35 with at least one comorbidity 5. Had given written informed consent	1. Weight loss 2. Sleeve volume 3. FT score 4. Weight Regain	"BSG had significantly lower sleeve volume, significantly lower WR, and significantly lower FT scores than LSG after four years from surgery; however, volume changes were not correlated with weight loss."
		LSG, 1279(90.65)			35.54 ± 10.84	323(25.3)		47.62 ± 7.05	1. HTN, 40(30.3) 2. DM, 24(18.2) 3. SA, 19(14.4) 4. Dyslipidemia, 40(30.3) 5. OA, 34(25.8) 6. CD, 8(6.1) 7. Psych. disorders, 16(12.1) 8. Vascular diseases, 20(15.2)			
7	Hany et al. 2023 [23]	LBSG, 25(50)	Egypt	RCT	40.7 ± 11.2	4(16)	Up to Two	47.7 ± 5	1. HTN, 8(32) 2. DM, 4(16) 3. Dyslipidemia, 7(28)	1. Patients who had undergone primary LSG 2. Age between 18 and 60 years 3. Had experienced weight regain exceeding their nadir 4. With a BMI more than 40 or 35 with at least one comorbidity 5. Had given written informed consent	1. BMI 2. EWL(%) 3. FT score	"Laparoscopic re-LSG is feasible and safe with satisfactory outcomes in patients with weight regain after LSG who have gastric dilatation without reflux esophagitis. Both groups had comparable significant weight loss effects and improvement of associated medical problems. The BLSG tends to have a more stable weight loss after two years with a significantly lower BMI, lower stomach volume, and less weight regain. Food tolerance decreased in both groups but reduced more in the BLSG group. After a 2-year follow-up, we may regard both procedures as safe, with no significant differences in the occurrence of complications and nutritional deficits."
		LSG, 25(50)			39.7 ± 8	8(32)		46.9 ± 6.1	1. HTN, 8(32) 2. DM, 6(24) 3. Dyslipidemia, 8(32)			
8	Bhandari et al. 2019 [20]	LBSG, 68(30.91)	India	Prospective Cohort Study	40.5 ± 12.6	37(55)	Up to Five	44.6 ± 8.3	HTN, 25(36.8)	1. All patients who underwent SG or BSG 2. Had undergone operations at our institution throughout the year 2012 3. Followed up for up to 5years 4. BMI (mean 44.9 ± 8.4 kg/m2 for the entire group) 5. Had given written informed consent	1. Weight Loss 2. BMI 3. Post-operative complications	"BSG is safe and produces substantially more weight loss than non-banded SG at 2 45 through 5 postoperative years, with minimal side effects."
		LSG, 152(69.09)			40.4 ± 13.2	71(47)		45.0 ± 8.4	HTN, 69(45.4)			
9	Fink et al. 2017 [27]	LBSG, 42(50)	Germany	Retrospective cohort study	40.05 ± 12.08	12(28.57)	Mean(Three)	54.93 ± 7.42	1. DM, 7(16.67) 2. HTN, 27(64.29)	1. All patients who underwent SG or BSG 2. Operated between November 2010 and October 2014 3. Followed up for up to 3 years	1. EWL(%) 2. Morbidity, Mortality and complications	"BLSG is a safe procedure showing similar comorbidity to conventional LSG. However, BLSG leads to a higher rate of postoperative regurgitation. Weight loss is significantly improved three years after surgery. Hence, additional Ring implantation might be an option for increased restriction in LSG surgery."
		LSG, 42(50)			42.21 ± 13.49	14(33.33)		53.46 ± 6.69	1. DM, 9(21.43) 2. HTN, 23(54.76)			
10	Fink et al. 2020 [28]	LBSG, 47(50)	Germany	RCT	43.3 ± 9.196	12(25.5)	Mean(Three)	51 ± 6.64	1. DM, 11(23.4) 2. HTN, 26(53.3) 3. GERD, 7(14.9) 4. Hiatal hernia, 9(19.1) 5. Esophagitis, 11(23.4)	1. Patient age ≥18 and ≤65 years 2. History of conservative weight-loss treatment failure 3. With a BMI more than 40 or 35 with at least one comorbidity 4. Had given written informed consent	1. EWL(%) 2. HA1C 3. Diabetes Remission	"BSG provided better weight loss than non-banded SG 3 years after surgery. Regurgitation was the main clinically relevant negative effect after BSG."
		LSG, 47(50)			40.9 ± 9.03	16(34)		50.7 ± 6.47	1. DM, 6(12.8) 2. HTN, 20(42.6) 3. GERD, 7(14.9) 4. Hiatal hernia, 6(12.8) 5. Esophagitis, 6(12.8)			
11	Gentileschi et.al 2020 [10]	LBSG, 25(50)	Italy	RCT	47.3 ± 6.58	9(36)	Up to Four	45.95 ± 5.85	1. DM, 5(20) 2. HTN, 7(28) 3. OSAS, 2(8)	1. Patient age ≥18 and ≤60 years 2. History of conservative weight-loss treatment failure 3. With a BMI more than 40 or 35 with at least one comorbidity 4. Had given written informed consent	1. BMI 2. EWL(%) 3. Post-operative complications	"LBSG is a safe procedure with no impact on postoperative complications. /e banded sleeve showed a significantly greater weight loss in the midterm follow-up. Considering the issue of Weight regain observed after LSG, the placement of a peri-gastric ring during the ﬁrst procedure may be a strategy to improve the results."
		LSG, 25(50)			43.7 ± 9.8	11(44)		47.3 ± 6.58	1. DM, 7(28) 2. HTN, 14(56) 3. OSAS, 6(24)			
12	Karcz et.al 2014 [25]	LBSG, 25(50)	Germany	Retrospective cohort study	Mean(42.6)	7(28)	Mean(0.9167)	Mean(56.1)	1. DM, 6(24) 2. HTN, 12(48)	1. From January to August 2012 2. Patients underwent BLSG at one institution 3. With a BMI more than 40 or 35 with at least one comorbidity 4. Had given written informed consent	1. BMI 2. EWL (%)	"Ring implantation does not increase the Duration of surgery or early surgical complications. Weight loss in the first follow-up year is not influenced, but the incidence of vomiting is raised after 12 months when patients start to increase eating volume."
		LSG, 25(50)			Mean(43.6)	8(32)		Mean(57)	1. DM, 6(24) 2. HTN, 15(60)			
13	Lemmens et.al 2018 [24]	LBSG, 96(65.31)	Belgium	Retrospective cohort study	47.9 ± 12.2	60(62.5)	Up to Five	43.7 ± 7.3	1. DM, 12(12.5) 2. HTN, 22(22.92) 3. SA, 32(33.33) 4. Hypercholesterolemia, 19(19.79) 4. Depression, 1(1.04)	1. Patients that had either NLSG or BLSG 2. Between May 2010 and July 2017 3. Follow-up visits took place at 3, 6, 12, 24, 36, 48, and 60 months post-operatively 4. Had given written informed consent	1. Weight Loss Failure 2. Weight Regain 3. Post-operative complications	"BLSG surgery was found to be safe and effective in maintaining weight loss in the long term compared to the NLSG group with a low incidence of band-related problems. Additionally, the NLSG group had a higher rate of weight loss failure and weight regain at five years compared to the BLSG group."
		LSG, 51(34.69)			54.8 ± 14.1	22(43.14)		44.9 ± 7	1. DM, 12(23.53) 2. HTN, 17(33.33) 3. SA, 13(25.49) 4. Hypercholesterolemia, 11(21.57) 4. Depression, 1(1.961)			
14	Soliman et al. 2015 [26]	LBSG, 24(50)	Egypt	Retrospective cohort study	33 ± 11	18(75)	1.25(SD 0.267)	39 to 56	1. DM, 2(8.33) 2. HTN, 4(16.67) 3. SA, 1(4.167)	1. From June 2011 to July 2012 2. Underwent BSG in which a strip of Gortex mesh 3. BMI] 39–56 kg/m2 4. The patients’ average age was 26 years 5. Had given a written informed consent	1. BMI 2. Weight Loss	"BSG provides better results in the curve of losing weight and weight loss maintenance. While this Study documents the feasibility and possible beneﬁts of this modiﬁcation, other prospective studies with long-term follow-up are needed to establish its role in surgical weight loss procedures."
		LSG, 24(50)			34 ± 10.5	12(50)			1. DM, 2(8.33) 2. HTN, 2(8.33) 3. SA, 1(4.167)			
15	Tognoni et al. 2016 [29]	LBSG, 25(50)	Italy	RCT	45.7 ± 12.7	9(36)	Mean(One)	45.99 ± 6.25	1. DM, 5(20) 2. HTN, 7(28) 3. OSAS, 2(8)	1. From June 2011 to July 2012 2. Patients were selected for SG or BSG 3. BMI Ranges from 39.74 to 53.61 4. Results obtained with 50 patients at 1-year follow-up 5. Had given a written informed consent	1. BMI 2. Post-operative complications	"The results of bodyweight loss did not document any statistically significant differences among the two groups, even though the LBSG group showed a mean BMI slightly lower than that of the control group."
		LSG, 25(50)			43.7 ± 9.8	9(36)		47.03 ± 6.58	1. DM, 7(28) 2. HTN, 14(56) 3. OSAS, 6(24)			

Risk of Bias Evaluation

**Figure 2:**
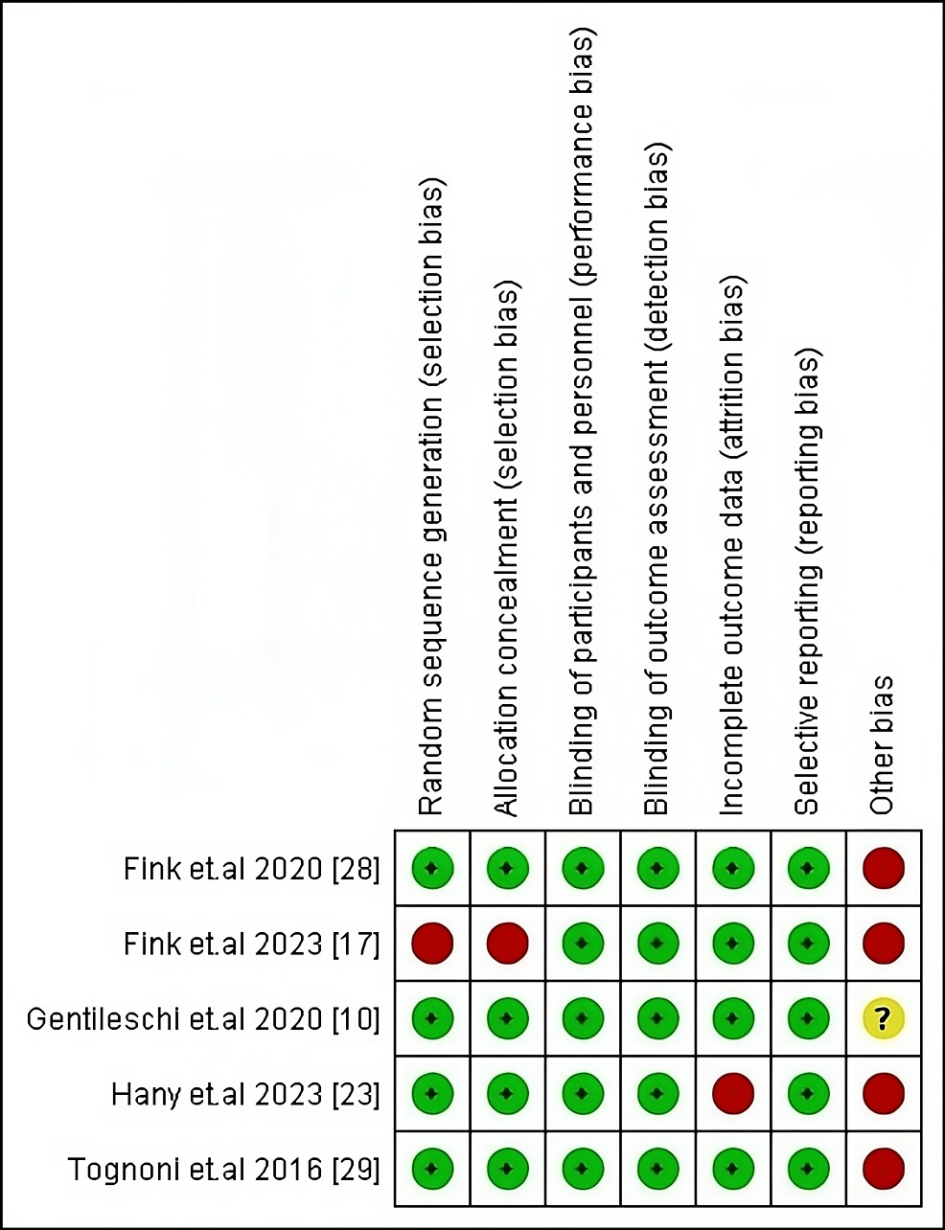
Risk of bias summary of the included RCTs RCT= Randomized clinical trial

Our five included RCTs were judged low risk regarding detection, performance, and reporting biases. Conversely, according to the domain of other potential biases, judged high or unclear risk. Fink et al. [17] had a high risk in selection, allocation, and other sources of bias. The risk of bias of included RCTs is summarized in Figure 2. Regarding our included cohort studies and according to the NIH tool. All of them were of fair quality, scoring between 8 and 10, except Albalkiny et al. [18], which had good quality. Similarly, our nonrandomized study judged good quality according to MINORS Criteria. The judgment tables of both are provided in Tables 2, 3, respectively.

**Table 2:** NIH quality assessment tool for observational cohort and cross-sectional studies NIH - National Institutes of Health

ID	NIH Quality Assessment Tool for Observational Cohort and Cross-Sectional Studies															Quality rating: Good (11-14) or Fair (7.5-10.5) or Poor (0-7), Yes = 1 // No = 0.5 // NR & NA & CD = 0
	1. Was the research question or objective in this paper clearly stated?	2. Were eligibility/selection criteria for the study population prespecified and clearly described?	3. Were the participants in the study representative of those who would be eligible for the test/service/intervention in the general or clinical population of interest?	4. Were all eligible participants that met the prespecified entry criteria enrolled?	5. Was the sample size sufficiently large to provide confidence in the findings?	6. For the analyses in this paper, were the exposure(s) of interest measured prior to the outcome(s) being measured?	7. Was the time frame sufficient so that one could reasonably expect to see an association between exposure and outcome if it existed?	8. For exposures that can vary in amount or level, did the study examine different levels of the exposure as related to the outcome (eg, categories of exposure, or exposure measured as continuous variable)?	9. Were the exposure measures (independent variables) clearly defined,valid, reliable, and implemented consistently across all study participants?	10. Was the exposure(s) assessed more than once over time?	11. Were the outcome measures prespecified, clearly defined, valid, reliable, and assessed consistently across all study participants?	12. Were the people assessing the outcomes blinded to the participants' exposures/ interventions?	13. Was the loss to follow-up after baseline 20% or less? Were those lost to follow-up accounted for in the analysis?	14. Were key potential confounding variables measured and adjusted statistically for their impact on the relationship between exposure(s) and outcome(s)?	Total scores	
	Yes / No / Not reported (NR) or cannot determine (CD) or not applicable (NA)	Yes / No / Not reported (NR) or cannot determine (CD) or not applicable (NA)	Yes / No / Not reported (NR) or cannot determine (CD) or not applicable (NA)	Yes / No / Not reported (NR) or cannot determine (CD) or not applicable (NA)	Yes / No / Not reported (NR) or cannot determine (CD) or not applicable (NA)	Yes / No / Not reported (NR) or cannot determine (CD) or not applicable (NA)	Yes / No / Not reported (NR) or cannot determine (CD) or not applicable (NA)	Yes / No / Not reported (NR) or cannot determine (CD) or not applicable (NA)	Yes / No / Not reported (NR) or cannot determine (CD) or not applicable (NA)	Yes / No / Not reported (NR) or cannot determine (CD) or not applicable (NA)	Yes / No / Not reported (NR) or cannot determine (CD) or not applicable (NA)	Yes / No / Not reported (NR) or cannot determine (CD) or not applicable (NA)	Yes / No / Not reported (NR) or cannot determine (CD) or not applicable (NA)	Yes / No / Not reported (NR) or cannot determine (CD) or not applicable (NA)		
Bhandari et.al 2019 [20]	Yes	Yes	Yes	Yes	NR	Yes	Yes	NA	Yes	NR	NR	NA	Yes	Yes	9	Fair
Fink et.al 2017 [27]	Yes	Yes	Yes	NR	NR	Yes	Yes	NA	Yes	NR	NR	NA	Yes	Yes	8	Fair
Karcz et.al 2014 [25]	Yes	Yes	Yes	NR	NR	Yes	Yes	NA	Yes	NR	NR	NA	Yes	Yes	8	Fair
Lemmens et.al 2018 [24]	Yes	Yes	Yes	Yes	No	Yes	Yes	NA	Yes	NR	NR	NA	Yes	Yes	9.5	Fair
Soliman et.al 2015 [26]	Yes	Yes	Yes	Yes	NR	Yes	Yes	NA	Yes	NR	Yes	NA	Yes	Yes	10	Fair
Afifi et.al 2022 [19]	Yes	Yes	Yes	Yes	NR	Yes	Yes	NA	Yes	NR	Yes	NA	Yes	Yes	10	Fair
Albalkiny et.al 2022 [18]	Yes	Yes	Yes	Yes	Yes	Yes	Yes	NA	Yes	NR	Yes	NA	Yes	Yes	11	Good
Hany et.al 2022 [22]	Yes	Yes	Yes	NR	Yes	Yes	Yes	NA	Yes	NR	NR	NA	Yes	Yes	9	Fair
Hany et.al 2022 (2) [30]	Yes	Yes	Yes	NR	Yes	Yes	Yes	NA	Yes	NR	Yes	NA	Yes	Yes	10	Fair

**Table 3:** MINORS criteria for assessment of non-randomized clinical trial

ID	MINORS criteria								
	1. A stated aim of the study?	2. Inclusion of consecutive patients?	3.Prospective collection of data?	4. Endpoint appropriate to the study aim?	5. Unbiased evaluation of endpoints?	6. Follow-up period appropriate to the major endpoint?	7. Loss to follow up not exceeding 5%?	Total scores: Yes = 1 // No = 0.5 // NR & NA & CD = 0	Quality rating: Good (5.5-7) or Fair (4-5.5) or Poor (3.5-0)
	Yes / No / Not reported (NR) or cannot determine (CD) or not applicable (NA)	Yes / No / Not reported (NR) or cannot determine (CD) or not applicable (NA)	Yes / No / Not reported (NR) or cannot determine (CD) or not applicable (NA)	Yes / No / Not reported (NR) or cannot determine (CD) or not applicable (NA)	Yes / No / Not reported (NR) or cannot determine (CD) or not applicable (NA)	Yes / No / Not reported (NR) or cannot determine (CD) or not applicable (NA)	Yes / No / Not reported (NR) or cannot determine (CD) or not applicable (NA)		
Fouly et.al 2022 [21]	Yes	NR	Yes	Yes	Yes	Yes	Yes	6	Good

Outcomes

Anthropometric Parameters

**Figure 3:**
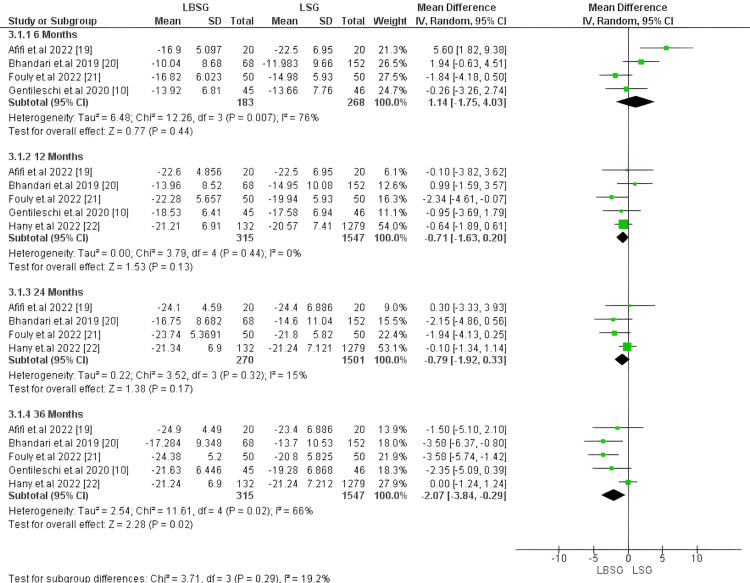
Forest plot of BMI

**Figure 4:**
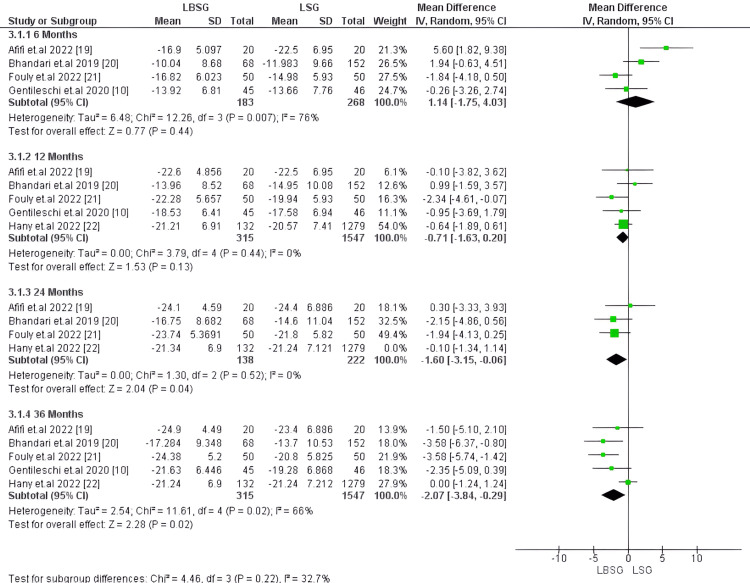
Forest plot of BMI (after resolving heterogeneity)

Body Mass Index (BMI): At six months, BMI was estimated only in four studies encompassing 451 patients [10,19-21] with no substantial difference between LBSG and LSG groups. The corresponding MD and 95%CI is 1.14 [-1.75,4.03], yielding a p-value of 0.44. Studies pooled for this outcome at this time were heterogenous with I2 and Chi2-p (76%, 0.007), respectively. However, at 12 months, BMI was compared in five studies, including 1,862 patients, with no substantial difference between the two groups -0.71 [-1.63, 0.20], p-value 0.13 [10,19-22]. Similarly, four studies including 1,771 patients at 24 months observed no substantial difference between LBSG and LSG groups with pooled MD, 95%CI, and p-value of -0.79, [-1.92, 0.33] and 0.17, respectively [19-22]. Our pooled studies at 12 and 24 months were homogenous with I2, Chi2-p (0%, 0.44) and (15%, 0.32), respectively. On the other hand, at 36 months, five studies involving 1862 patients reported substantially lower BMI: MD -2.07 [-3.84, -0.29], p = 0.02 [10,19-22]. Studies pooled for this outcome were heterogenous with Chi2-p = 0.02 and I2 = 66%; however, we could resolve heterogeneity by excluding Hany et al. [22] (Chi2-p = 0.72 and I2 = 0%). Still, the pooled MD substantially favored LBSG (-2.99 and 95%CI [-4.34, -1.65]); p-value <0.0001). Figures 3, 4, respectively illustrate the forest plots for this outcome.

**Figure 5:**
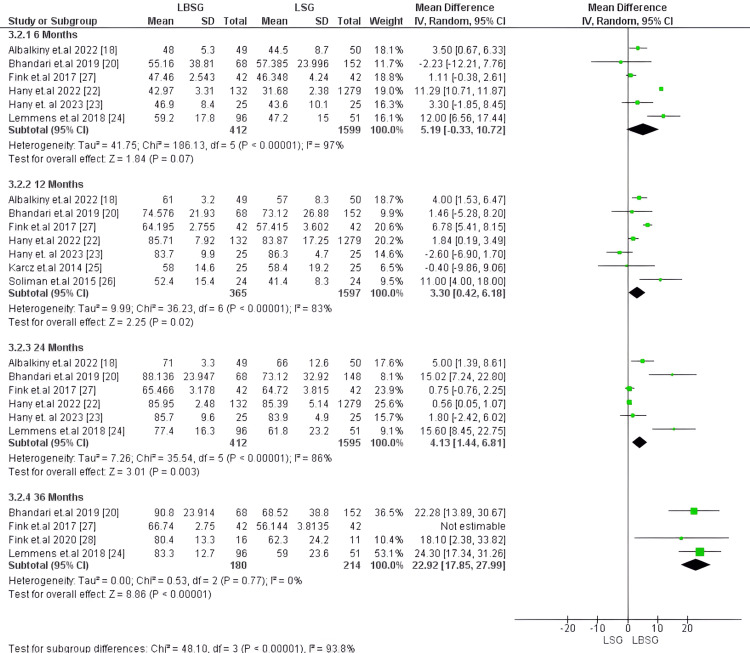
Forest plot of excess weight loss (EWL), %

**Figure 6:**
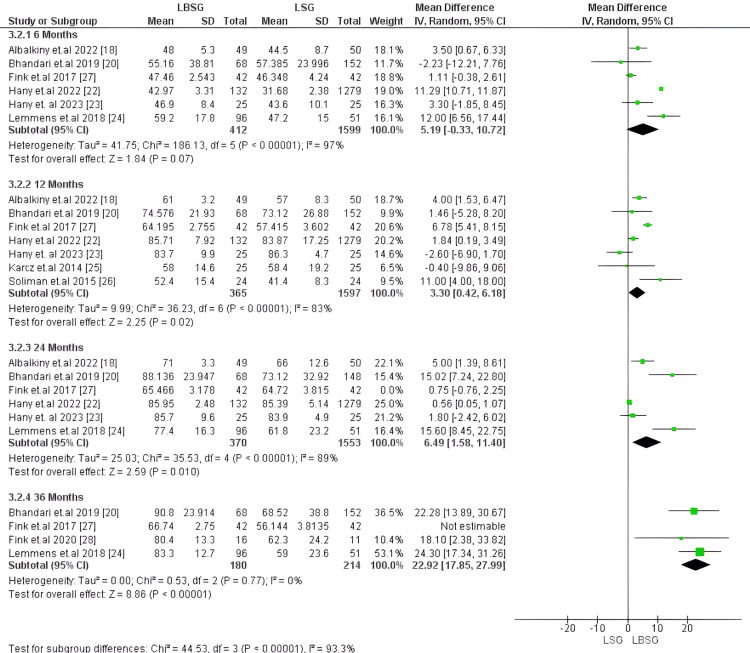
Forest plot of excess weight loss (EWL), % (after resolving heterogeneity)

Excess weight loss (EWL), %: EWL was evaluated at four time points: 1) after six months of follow-up, it was compared in six studies encompassing 2,011 patients with no substantial difference in EWL between LBSG and LSG groups with MD of 5.19 [-0.33, 10.72]; p-value of 0.07 [17,18,20,22-24]. 2) After 12 months, seven studies, including 1,962 patients, reported this outcome with substantially greater EWL for the LBSG group with 3.30 [0.42, 6.18], the p-value was 0.02. 3) EWL was reported after 24 months of follow-up in six studies encompassing 2,007 patients [17,18,20,22-24]. our analysis showed substantially higher %EWL in the LBSG group than the other group (MD=4.13; 95%CI = [1.44, 6.81]; p-value of 0.003). there was a major heterogeneity among the pooled studies at these points of follow-up six, 12, and 24 months with corresponding (I2, Chi2-p) of (97%, <0.00001), (83%, <0.00001) and (86%, <0.00001), respectively. EWL after 36 months was reported in four studies involving 478 patients. Our analysis showed that the LBSG group had substantially greater %EWL than the other group (MD = 18.43 [9.44, 27.42]; p-value<0.00001). However, the studies were heterogenous, but we could resolve heterogeneity after excluding Fink et al. [27] (I2 = 0%, p =0.77). The pooled MD also favored LBSG (22.92 [17.85, 27.99]; p-value<0.00001). Figures 5, 6, respectively, depict the forest plot for %EWL.

Operative Time

**Figure 7:**
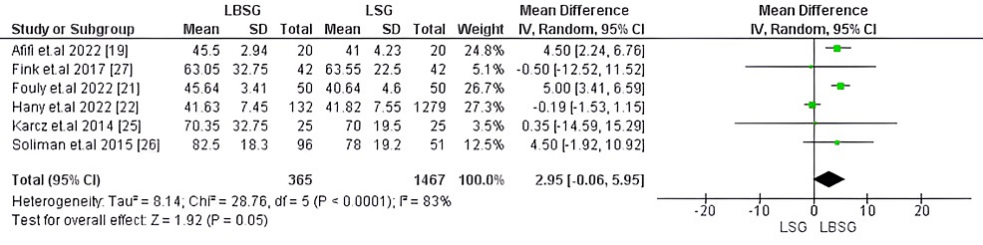
Forest plot of operative time (min)

Mean operative time in minutes was assessed in six studies, including a total of 1,832 patients [19,21,22,25-27]. Our analysis showed no substantial difference in operative time between LBSG and LSG groups (MD was 2.95, 95%CI [-0.06, 5.95]; p-value= 0.05). Pooled studies were heterogenous with I2 83% and Chi2-p <0.0001 (Figure 7).

Resolution of Comorbidities

**Figure 8:**
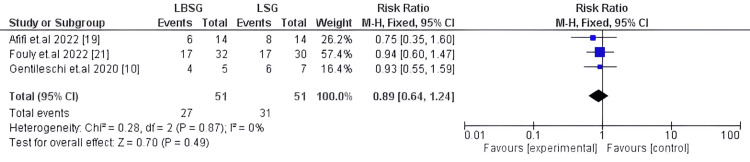
Forest plot of resolution of comorbidities

Three studies with 102 patients investigated this outcome during the post-operative follow-up, resulting in no substantial difference between both procedures (LBSG and LSG) [10,19,21]. Our pooled proportion (RR: 0.89, 95%CI: [0.64, 1.24]) and p-value =0.49, the pooled studies exhibited homogeneity I2 0%, Chi2-p=0.87. The forest plot is illustrated in Figure 8.

Overall Complications

**Figure 9:**
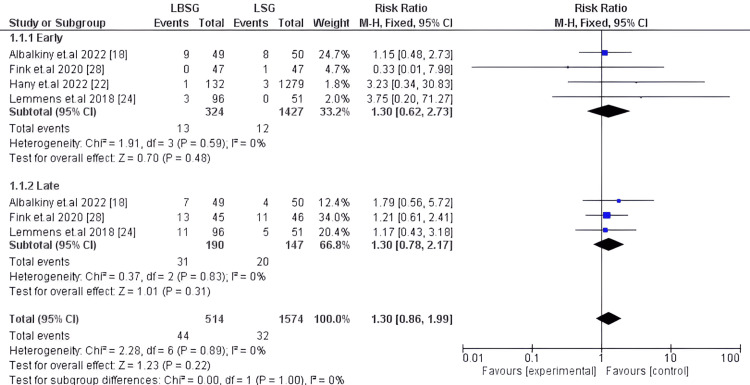
Forest plot of overall complications

Post-operative overall complications were studied in four studies totaling 2,088 participants [18,22,24,28]; the results showed insignificant differences between LBSG and LSG, pooled RR;95%CI: 1.30 [0.86, 1.99], p-value=0.22. Regarding early complications, it was evaluated in four studies, including only 1,751 patients [18,22,24,28]. Also, LBSG did not substantially differ from LSG; our pooled analysis estimated (RR: 1.30, 95%CI: [0.62, 2.73]) p-value=0.48. However, three articles included 337 patients [18,24,28] who reported late complications, and our provided results did not show any substantial difference between the two groups (RR: 1.30, 95%CI: [0.78, 2.17]) p-value=0.31. Our pooled studies for early, late, and overall complications showed homogeneity; hence, it was analyzed using a fixed effect model; the corresponding I2 and Chi2-p were (0%; 0.59), (0%; 0.83), and (0%; 0.89) respectively. Figure 9 shows the forest plot demonstrated post-operative complications.

Post-operative Bleeding

**Figure 10:**
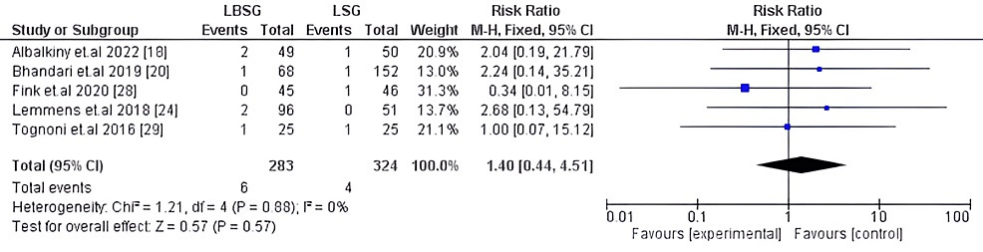
Forest plot of post-operative bleeding

Six hundred-seven patients in five studies [18,20,24,28,29] reported post-operative bleeding. Our pooled proportion showed a statistically insignificant difference between LBSG and LSG groups with RR: 1.40 [0.44, 4.51] and p-value=0.57. The studies were homogenous and analyzed using fixed model I2 0%; Chi2-p=0.88. Figure 10 depicts the forest plot for this outcome.

Reflux

**Figure 11:**
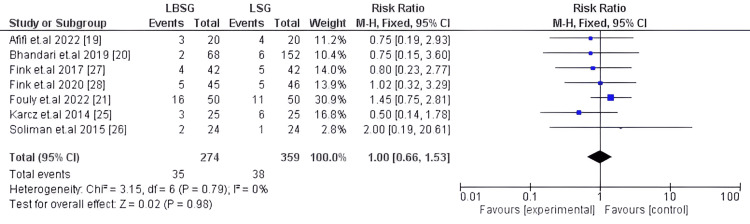
Forest plot of reflux incidence

Regarding post-operative reflux symptoms, it was evaluated in seven studies encompassing 633 patients [19-21,25,26,26,28] with statistically insignificant differences between the compared interventions (LBSG and LSG). Our pooled RR and its corresponding 95%CI were 1.00 [0.66, 1.53], p-value=0.98. Studies pooled for this outcome were homogenous with I2 0% Chi2-p=0.79. Figure 11 illustrates the plot for post-operative reflux.

Regurgitation

**Figure 12:**
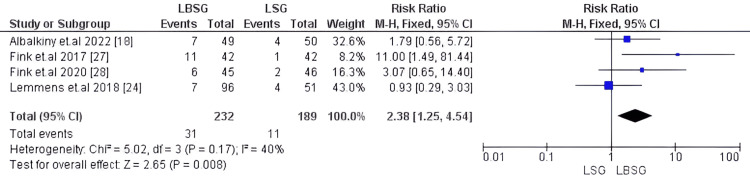
Forest plot of regurgitation incidence

Four studies, including 421 patients [18,24,27,28], evaluated post-operative regurgitation; our analysis showed substantially higher regurgitation symptoms in the LBSG group when compared to the LSG our RR: 2.38 [1.25, 4.54] and p-value=0.008. There was substantial heterogeneity among the pooled studies with 40%, 0.17. Figure 12 shows the forest plot for this outcome.

Discussion

Our systematic review and meta-analysis encompassed 15 studies, including 3,929 patients. After three years of follow-up, the banded group exhibited a significantly lower BMI (p-value <0.0001) and a notably higher percentage of EWL at multiple time intervals (one, two, and three years), with the most pronounced difference observed at the three-year (p-value <0.00001). Additionally, the LBSG group significantly reduced the incidence of post-operative regurgitation symptoms (p-value = 0.008) compared to the non-banded LSG group. However, we did not identify a substantial difference between the two groups regarding BMI after one year, %EWL after six months, operative time, post-operative bleeding, reflux, or overall complication.

The superiority of the banded procedure over traditional LSG stems from its potential to address a primary concern in LSG outcomes: post-operative weight regain, which remains a predominant cause of LSG failure and subsequent revisional surgeries. This weight regain often correlates with gastric pouch dilatation. Introducing a band around the gastric tube aims to mitigate this issue by preserving the size of the gastric pouch, thereby potentially reducing the incidence of post-operative weight regain, a critical factor contributing to improved long-term outcomes in banded procedures compared to traditional LSG [7]. Previous literature revealed that inserting a nonadjustable gastric band improved weight loss outcomes in various bariatric operations [31-35].

The potential superiority of the banded procedure may be attributed to the band's impact on reducing appetite and activating peripheral satiety mechanisms. This effect involves slowing down the transmission of food within the longitudinal part of the sleeve, exerting minimal restriction. In contrast, LSG primarily operates through size reduction with minimal influence on satiety. Therefore, LBSG is hypothesized to enhance weight loss by combining both mechanisms, potentially amplifying weight loss outcomes without introducing additional complications [36,37].

Previous studies have posited that extended operative durations, particularly exceeding four hours, could potentially lead to prolonged hospital stays and an increased likelihood of post-operative complications. Moreover, prolonged operative times might negatively impact the recovery process of patients undergoing surgery [38-41]. This aligned with our findings, indicating that banded LBSG exhibits a notably shorter operative time than traditional LSG. This disparity in duration might signify the relatively simpler technical approach associated with LBSG [6]. In the literature, LSG was linked to aggravated preexisting regurgitation symptoms. This may be due to the anatomic and physiological changes caused by LSG [42,43]. However, our study reported an increase in regurgitation with the LBSG group.

Chaouch et al.'s meta-analysis, encompassing 753 patients and comparing LBSG and LSG, concluded that banding in sleeve gastrectomy may result in a lower BMI and a higher %EWL after one year of follow-up. Additionally, the study noted a significant reduction in %EWL after both one year and three years of follow-up in the banded group. However, the analysis found no substantial evidence supporting the superiority of LBSG over LSG in terms of mitigating vomiting, de novo gastroesophageal reflux disease (GERD), food intolerance, or reducing operative time [11]. This aligned with our findings regarding BMI, %EWL, reflux, and operative time. Still, our evidence was more powered with a larger sample size and more robust stratifications according to follow-up periods.

Contrary to previous reflux findings, Alexander et al. presented a substantial reduction in symptoms following LBSG. Among 15 patients with preoperative reflux, eight experienced complete resolution of reflux symptoms, while the remaining patients observed symptomatic improvement. However, three patients developed mild reflux symptoms de novo after undergoing LBSG. Notably, none of the patients in their study necessitated reoperation for band removal due to reflux-related issues [44]. In a systematic review conducted by Gehrer et al., the authors concluded that the data regarding the impact of LSG on GERD were inconclusive [45]. Among the included studies, four demonstrated an elevated incidence of GERD post-operatively, while seven indicated a decrease in GERD following the procedure. However, it is important to note that all studies indicating a disparity in reflux symptoms between the studied procedures had a notably limited sample size or single-arm design, potentially impacting the robustness and generalizability of their findings.

Furthermore, previous literature supported our findings as follows. Lemmens et al., conducted over a five-year follow-up period, revealed a stark contrast in weight regain between LBSG and LSG groups [24]. The findings showcased a significantly lower incidence of weight regain in the LBSG cohort, standing at 2%, in contrast to a substantially higher proportion of 19.6% observed in the LSG group. These results were further supported by evidence indicating that removal of the band led to subsequent weight gain, corroborating the pivotal role of banding in preventing weight regain following sleeve gastrectomy procedures [46]. On the other hand, Tognoni et al. noted no statistically significant variances between the non-banded and banded groups during a 12-month follow-up. However, they observed slightly lower mean BMI values within the LBSG cohort than in the LSG group [29]. Similarly, in a matched-cohort analysis encompassing 50 patients evenly distributed between both study arms, Karcz et al. did not discern statistically substantial distinctions in the percentage of estimated weight loss at the 12-month milestone between the two groups [25].

Parmar et al. reviewed six studies with 236 LBSG patients, finding a 77.4% mean weight loss at 12 months, 11.8% complications, 0.85% mortality, and 5.5% reoperations. However, they were limited by long-term data, which hindered definitive conclusions, prompting a call for more extensive studies for clearer insights [42]. Gentileschi et al. randomized 50 patients into banded and non-banded LSG groups over four years, finding banded LSG yielded higher weight loss without added risks [9].

Although pros regarding LBSG were mentioned, there have been notable concerns surrounding the use of banding devices in bariatric surgery, primarily due to the potential risks associated with displacement, erosion, or slippage. These concerns have emerged based on previous data associated with using AGB. This procedure led to many patients requiring revision surgery to address complications stemming from the banding device [47]. Unlike the AGB, the band used in LBSG is a relatively thin ring that remains loosely positioned without applying pressure on the sleeve. Importantly, its application involves minimal dissection on the lesser omentum, distinguishing it from the approach used in AGB procedures [9].

Currently, the number of cases involving banded LBSG remains relatively restricted, particularly when compared to the volume of other interventions within the field of bariatric surgery [18]. Nonetheless, a meta-analysis encompassing over 8,000 patients who underwent banded Roux-en-Y gastric bypass (RYGB) with a decade-long follow-up revealed remarkably low complication rates (2.3% for erosion and 1.5% for slippage) [48]. However, it is essential to note that this analysis was a single-arm study lacking comparability, potentially impacting its inferential statistical power.

Our study represents an updated evaluation of the comparative efficacy between banded and non-banded LSG, featuring a larger sample size and an extended follow-up duration of up to three years, allowing for a more comprehensive assessment of outcomes. However, it is important to emphasize the necessity for further extended follow-up periods to monitor the emergence of potential complications such as perforation, migration, or erosion. These complications may manifest over a longer duration post-surgery [49].

Still, our study was not free of limitations as follows. Firstly, including diverse study designs introduces potential heterogeneity, impacting the coherence and generalizability of our findings. Moreover, underpowered studies with small sample sizes might compromise our meta-analysis's statistical power and precision. Additionally, confounding factors in baseline characteristics, notably variations in BMI and other comorbidities across studies, could have influenced observed outcomes. Lastly, the largest study in our analysis featured unequal arms (LBSG vs. LSG) with a substantial ratio disparity (1:10), potentially introducing bias and impacting the comparative analysis [22]. These limitations underscore the need for caution when interpreting our study's findings. The restricted sample sizes, potential for longer-term complications, and the complexity of outcomes like the resolution of comorbidities warrant careful consideration and moderation in drawing definitive conclusions from our results.

## Conclusions

In our study, LBSG showed a substantial decrease in BMI at three-year follow-up and higher %EWL at one, two, and three years' time points. However, LBSG procedures exhibited a higher incidence of post-operative regurgitation symptoms than LSG. Still, no substantial differences emerged in BMI at six, 12, and 24 months, %EWL at six months, operative time, bleeding, reflux, or overall complications. Further high-quality studies should address the limitations mentioned in our study, emphasizing larger sample sizes, uniform study designs, and extended follow-ups to evaluate outcomes in banded versus non-banded LSG comprehensively.
